# Treatment of Alzheimer’s disease by combination of acupuncture and Chinese medicine based on pathophysiological mechanism: A review

**DOI:** 10.1097/MD.0000000000032218

**Published:** 2022-12-09

**Authors:** Zhao Liu, Ruiqian Guan, Fan Bu, Limin Pan

**Affiliations:** a Heilongjiang University of Traditional Chinese Medicine, Harbin, Heilongjiang Province, China; b Second Affiliated Hospital of Heilongjiang University of Traditional Chinese Medicine, Harbin, Heilongjiang Province, China; c First Affiliated Hospital of Heilongjiang University of Traditional Chinese Medicine, Harbin, Heilongjiang Province, China.

**Keywords:** Alzheimer’s disease, biomarkers, etiology, TCM syndrome

## Abstract

Alzheimer’s disease (AD) is a neurodegenerative disorder characterized by neurodegeneration, nerve loss, neurofibrillary tangles, and Aβ plaques. In modern medical science, there has been a serious obstacle to the effective treatment of AD. At present, there is no clinically proven and effective western medicine treatment for AD. The reason is that the etiology of AD is not yet fully understood. In 2018, the international community put forward a purely biological definition of AD, but soon this view of biomarkers was widely questioned, because the so-called AD biomarkers are shared with other neurological diseases, the diagnostic accuracy is low, and they face various challenges in the process of clinical diagnosis and treatment. Nowadays, scholars increasingly regard AD as the result of multimechanism and multicenter interaction. Because there is no exact Western medicine treatment for AD, the times call for the comprehensive treatment of AD in traditional Chinese medicine (TCM). AD belongs to the category of “dull disease” in TCM. For thousands of years, TCM has accumulated a lot of relevant treatment experience in the process of diagnosis and treatment. TCM, acupuncture, and the combination of acupuncture and medicine all play an important role in the treatment of AD. Based on the research progress of modern medicine on the pathophysiology of AD, this paper discusses the treatment of this disease with the combination of acupuncture and medicine.

## 1. Introduction

Alzheimer’s disease (AD) has been declared as a “global public health priority” by the World Health Organization. Accumulation of plaques and tangles in the human brain is a pathological feature of AD, but it has long been recognized that these symptoms occur in other diseases, including amyloid cerebrovascular disease, TDP-43 content, and Lewy Body Disease.^[1]^ In 2018, National Institute on Aging (NIA) and the Alzheimer’s Association for Alzheimer’s Disease (AA) proposed a framework for the study of amyloid, Tau, and neurodegeneration (ATN) for the definition and diagnosis of AD,^[2]^ which enabled AD to move from a clinical-biological diagnosis to a purely biological definition applicable to both asymptomatic and symptomatic stages. However, the purely biological definition of AD itself has limitations, and the NIA-AA standard in 2018^[2,3]^ has caused a lot of debate regarding the use of biomarkers for the diagnosis of AD and the use of clinical signs, symptoms, and phenotypes.^[4,5]^ Therefore, nearly 4 years after the introduction of the NIA-AA criteria, the academic community began to reassess methods of diagnosing AD based on biomarkers.

AD is the leading cause of dementia in people over 65 years of age, and approximately 50% to 75% of people with dementia have AD. According to statistics collected worldwide, women are more likely to develop AD than men, and the risk increases with age.^[6,7]^ Patients with cardiovascular and cerebrovascular diseases, hypertension, and diabetes also have a higher risk of developing AD,^[8]^ which may explain the increase in AD cases in developing countries due to people’s lifestyles.^[9]^

**Figure 1.**
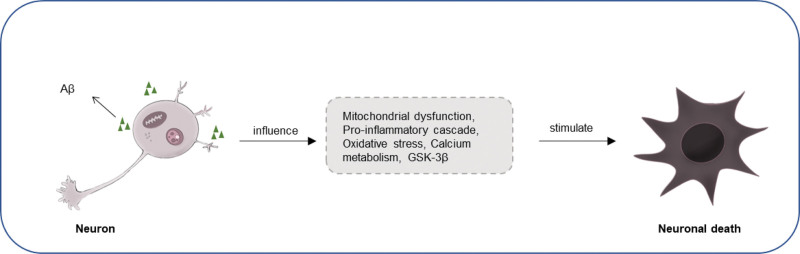
The presence of Aβ40 polymer and Tau fibrils constitute the main pathological condition of Alzheimer’s Disease (AD).

The presence of Aβ40 (Fig. 1) polymers and Tau fibrils constitutes the main pathological condition of AD; however, their association with any neurodegenerative disease is not clear, with pathological changes occurring long before the actual onset of symptoms. Various medical approaches to these pathological processes have proven unsuccessful in preclinical or clinical trials, mainly because of the low bioavailability, blood-brain barrier permeability, cell imperfection, and low half-life of drugs in current Western medicine treatment.^[10]^ Therefore, there must be new ways to treat diseases, the intervention of TCM (TCM), and the treasure house of TCM to help us cope with AD.

Because of the limitations of biomarkers, to date, there are only good concepts and hypotheses about the etiology and drug targets of AD, and there is no decisive theory that can be widely recognized by the academic community. Therefore, this article will introduce the etiology and diagnostic markers of AD and explore the possibility of syndrome differentiation and treatment of this disease through a combination of TCM and acupuncture.

## 2. Etiology and biomarkers of AD

### 2.1. Etiology of AD (Fig. 2)

Currently, there is still a lack of a reasonable time sequence for the occurrence of AD. Regarding the etiology of AD, the interaction between oligomers of Aβ protein and glial cells and neurons leads to various pathological and physiological abnormalities. These include mitochondrial dysfunction, stimulation of the pro-inflammatory cascade, tau phosphorylation, increased oxidative stress, dysregulation of calcium metabolism, enhanced glycogen synthase kinase-3β activity, stimulation of cell death, and neuronal apoptosis.^[11,12]^

**Figure 2.**
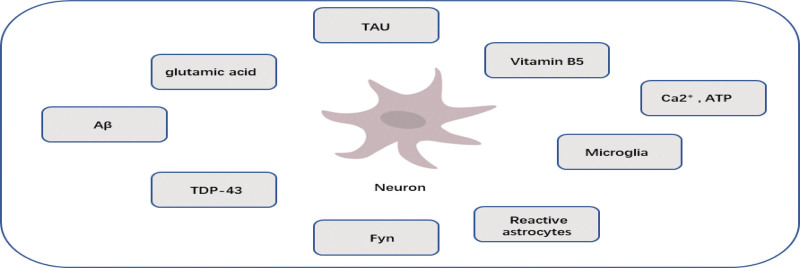
Etiology of Alzheimer’s Disease (AD).

### 2.2. Inheritance

Approximately 0.1% of AD cases are caused by genetics, which affects people who develop symptoms between the ages of 30 and 50 years. This is due to the autosomal dominance of one of the 3 genes encoding presenillins 1 and 2 and the amyloid precursor protein (APP). Mutations in any of these genes result in erroneous cleavage of APP to form Aβ42 instead of Aβ40.^[13]^ The accumulation and aggregation of Aβ42 forms senile plaques, which are one of the main causes of AD. ABCA7 and SORL1 are other autosomal dominant genes responsible for inherited AD.^[14]^ However, only a small proportion of AD cases exhibit autosomal dominant inheritance. Differences in genetic predisposition and environmental factors constitute a risk factor in most cases, with the presence of the ε4 allele of apolipoprotein E (APOE ε4) in heterozygotes and homozygotes increasing the risk by 3% and 15%, respectively. TREM2 is another gene involved in increasing the risk of AD when white blood cells do not control the amount of Aβ40 in the brain due to some variation in the allele of this gene. ABCA7, BIN1, CASS4, CD2AP, CELF1, CLU, CR1, EPHA1, FERMT2, HLA-DRB5, INPP5D, MEF2C, MS4A, NMES, PICALM, PTK2B, S1C24A4, SORL1, and ZCWPW1. A total of 19 genes were reported by the Genome Wide Association Study to clearly influence risk.^[15]^

### 2.3. Cholinergic hypothesis

The most widely accepted hypothesis states that the cause of AD is the lack of the adequate neurotransmitter acetylcholine (ACH) in neurons and neuromuscular regions.^[16]^ Biopsy reports from AD cases show that the activity of ACH transferase is decreased and the synthesis, absorption, and release of ACH are reduced. The reason for the deficiency of ACH in the brain is that its synthesis is reduced or the enzymatic activity of acetylcholin esterase is reduced. Four of the 5 main drugs marketed for the treatment of AD -- donepezil, rivastigmine, galantamine, and tacrine -- are based on this hypothesis.^[17]^

### 2.4. Amyloid hypothesis

This hypothesis suggests that the extracellular accumulation of Aβ42 inhibits the uninterrupted transmission of nerve impulses. Mutations in the APP gene, which is present on chromosome 21, are responsible for producing Aβ42, but not Aβ40. This form of amyloid beta protein is insoluble and forms aggregates, which, in turn, form cellulose. APOE decomposes Aβ, but in the presence of its allotrope, APOE ε4, Aβ does not decompose and comes together. Aβ oligomers bind to the prion protein receptor (CD230) on neurons, interfering with impulse transmission. Another possibility behind the disruption of neuronal communication is N-APP (the N-terminal part of APP), which binds to Death Receptor 6 on neurons.^[10]^

APP is a transmembrane protein that is cleaved by β-secretase and γ-secretase to form Aβ42. This form of protein is incorrectly folded, forming insoluble protein aggregates known as senile plaques, which cause toxicity. Under normal conditions, APP is cleaved by α-secretase and γ-secretase to form the soluble form of Aβ40 residue. The hyperphosphorylation of Tau protein is also the result of alterations in the protein kinase- and phosphatase-related pathways. The intracellular Ca2 + balance is disturbed, resulting in oxidative stress, and cell apoptosis is guided by mitochondrial signaling pathways. Plaques activate astrocytes and microglia and eventually kill nerve cells.^[18–20]^

### 2.5. Tau

Tau is phosphorylated at 3 different positions, threonine, serine, and an amino acid residue adjacent to proline. It attaches to microtubules at these residues. Protein hyperphosphorylation occurs mainly at T231, S235, and S265. Thereafter, proteins lose their ability to attach to microtubules and form neurofibrillary tangles.^[21]^ Neurofibrillary tangles formed by the aggregation of hyperphosphorylated tau threads disrupt the microtubule network of nerve cells. This leads to the inhibition of any biochemical communication between the inside and outside of the cell and destruction of the cytoskeleton. Although neurofibrillary tangles constitute one of the main pathophysiological conditions of AD, some studies have shown that inhibition of neurofibrillary tangles alone cannot be a potential target; therefore, any therapeutic approach must be multi-targeted to make a real difference in the treatment of AD, and tau-targeted drugs often target amyloid plaques as well.^[22,23]^

### 2.6. Excitotoxicity

Glutamate is another neurotransmitter present in the brain, and excessive glutamate levels can cause excitotoxicity, eventually leading to cell death. Minocycline is the only commercialized drug that targets the glutamate system to treat AD. It is a noncompetitive N-methyl-D-aspartate (NMDA) receptor antagonist that controls glutamate overstimulation. It regulates Ca2 + influx and efflux at the cell membrane via NMDA receptors. Minocycline also acts as an antagonist of the nACh and 5-HT3 receptors. Inhibition of NMDA can inhibit the expression of α-secretase, thereby reducing the production of Aβ.^[21,24]^

### 2.7. Vitamin B5 deficiency

Some studies have investigated the role of vitamin B5 in AD.^[25]^ Studies have found that the main and acute deficiency of vitamin B5 occurs in AD, and acute deficiency is considered to occur in AD. Areas thought to present with acute brain injury, including the middle temporal gyrus, entorhinal cortex, and hippocampus, experience more severe vitamin B5 deficiencies. Vitamin B5 is the precursor of acetyl-CoA and indirectly plays a key role in the metabolism of all organs, including the brain. Acetyl-Coa, the precursor of the neurotransmitter ACH, as well as complex fatty acyl groups that regulate the important insulating function of the myelin sheath, malfunctions in AD. Additionally, the concentration of vitamin B5 in the brain region coexists throughout the white matter. Therefore, researchers speculate that vitamin B5 deficiency may produce AD and ATN, which can be avoided or even reversed in the initial stage by oral administration of appropriate doses of vitamin B5.^[26]^

### 2.8. Mitochondrial cascade

Various mitochondrial factors decline with aging, leading to massive functional failure associated with the initiation of ATN, and the precise timing of these events is not clear.^[27]^ Therefore, some researchers have considered mitochondria to be therapeutic targets for nervous system diseases.^[28]^ Mitochondria are strictly required by neurons, mainly at breakouts, where they produce buffered Ca2 + and ATP. This is the basic process that generates membrane potential and nerve conduction along the axons.^[29]^ This confirms the high concentration of mitochondria at the synapse. A 20% to 30% reduction in glucose metabolism has been observed in AD-affected areas, and these changes occur long before the onset of pathophysiological symptoms associated with the disease.^[30]^ Some mitochondrial functions are severely affected in the AD brain,^[31]^ including mitochondrial morphology and number, oxidative phosphorylation, reactive oxygen species (ROS) production, Ca2 + buffering,^[32]^ mitochondria-ER contact sites,^[33]^ mitochondrial DNA mutation and oxidation,^[34]^ mitochondrial biogenesis, mitosis, and mitochondrial transport along the axon.^[35]^

The initial stage of AD, mild cognitive impairment (MCI), is characterized by a massive accumulation of oxidative stress markers such as protein oxidation and lipid peroxidation products, and a decrease in antioxidants in the brain.^[36]^ In the brains of patients with AD, there is a decline in activities related to mitochondrial energy production, such as alanine dehydrogenase complex, complex IV cytochrome C oxidase, mitochondrial isocitrate dehydrogenase, ATP synthase complex, and α-ketoglutarate dehydrogenase. In contrast, the activities of malate dehydrogenase and succinate dehydrogenase (complex II) were enhanced. ^[37]^ This results in the loss of control over the maintenance of mitochondrial inner membrane potential and mitochondrial ATP production.^[38]^ Further information on clinical trials of mitochondria in AD has been briefly presented.^[39]^

### 2.9. TDP-43 in AD

Reactive DNA-binding proteins (TDP-43) have been described as frontotemporal lobar degeneration with frontotemporal lobar degeneration with ubiquitin-positive Inclusions (FILD-U) and amyotrophic lateral sclerosis. TDP-43 residues in the brains of AD patients have been identified by various analyses and are closely related to the clinical phenotype of AD.^[40]^ Immunofluorescence studies have shown that more people have TDP-43 or phospho-TDP-43 in the middle than people with no cognitive impairment and MCI.^[41]^ A typical TDP-43 is typically stained in approximately 18% of cases when found pathologically in the hippocampus, and this number increases to 35% when it progresses to mental deterioration and to 46% of AD patients when pathologically examined.^[42]^ It binds to RNA-encoding genes involved in synapses and regulates the accumulation and endocytosis of TDP-43 near the end of axons. Overexpression of TDP-43 activates glycogen synthase kinase-3β, perturbing the balance of the cell and the interaction between this protein.^[43]^ TDP-43 is cleaved by calpain at its C-terminus to produce an N-terminal fragment that aggregates easily. Calpain is a Ca2+ -dependent serine protease. This results in the atypical aggregation of TDP-43 in the brain space.^[44]^

Notably, the intracellular domains of Aβ and APP (AICD) have been shown to initiate AD pathology with or without Aβ, and TDP-43 co-localizes with AICD in the nucleus, contributing to the upregulation of p53 mRNA. It also stimulates apoptosis induced by AICD.^[45]^

### 2.10. Fyn

Fyn has been reported to promote inhibition of Aβ oligomer-induced synaptic plasticity in vitro.^[46]^ Overexpression of Fyn in rodents accelerates the onset of cognitive impairment and the loss of prominence, whereas blocking Fyn expression inhibits synaptic loss. Histological studies of brain sections from patients with AD have shown a higher degree of Fyn staining in hyperphosphorylated neurons.^[47]^

Activation of tyrosine kinases by NMDAR-promoted calcium influx is required for intact long-term potentiation (LTP).^[48]^ A major study in mice with mutations in the non-receptor tyrosine kinases Fyn, Src, Abl, and Yes showed that Fyn has an important regulatory function in LTP induction.^[49]^ The NMDAR subunits NR2A and NR2B are phosphorylated by activated Fyn, but membrane stabilization and increased trafficking selectivity of NR2B result in enhanced transmission of the receptor and improved synaptic expression.^[50]^ Following these results, the activation of NR2B at Tyr1472 was clearly enhanced in Fyn-overexpressing rodents. At the site of Fyn, NR2B is regulated through its association with Postsynaptic Density Protein 95, a synaptic scaffold protein,^[51]^ and Aβ certainly does not affect LTP in the absence of Tau expression. Impairments in synaptic function and spatial memory were reversed. In the absence of tau protein overexpression, Fyn dissociates from NMDARs, and Aβ-induced synaptic toxicity is avoided. As existing evidence shows, not only the presence of Tau protein, but also its phosphorylated residues regulate the interaction of Fyn, Tau, NMDAR, and postsynaptic density protein 95.^[52]^

### 2.11. Reactive astrocyte

Reactive astrocytes are an early sign of AD and play both beneficial and detrimental roles in the pathogenesis of AD. The clearance of Aβ oligomers and aggregates is considered a positive response by astrocytes. Therefore, drugs that reduce Aβ toxicity by inducing autophagy or the ubiquitin system or that activate the antioxidant system, such as nuclear factor (erythrocyte-derived 2) transcription in astrocytes, could have therapeutic potential for AD. However, this may lead to metabolic changes in neurons that overproduce toxic metabolites, ROS, and/or inhibitory transmitters. These factors accumulate and chronically alter the properties of astrocytes, thereby rendering them neurotoxic. Therefore, stimulation of antioxidant systems and ROS-producing metabolites should be appropriately adjusted to adhere to a suitable oxidative state. In addition, manipulating the activity of MAO-B to regulate the GABA level of astrocytes could repair the cognitive impairment of AD.^[53]^

### 2.12. Role of microglia in the pathogenesis of AD

Microglia play a dual role in AD pathogenesis. On the one hand, microglia contribute to the pathogenesis of Aβ production and aggregation into oligomeric AD by releasing inflammatory mediators such as complement components, chemokines, free radicals, and inflammatory cytokines.^[54]^ Microglia, on the other hand, produce anti-Aβ antibodies and stimulate the elimination of amyloid plaques. Neurons and glial cells have many enzymes that degrade Aβ, such as insulin-degrading enzymes, nephrolytic enzymes, endothelin-converting enzyme I, and angiotensin-converting enzyme. Neuroinflammation caused by microglial activation represents a vicious cycle. Aβ plaques activate microglia, which produce inflammatory mediators, leading to inflammation and the accumulation of Aβ.^[55]^ Therefore, inhibiting this cycle by preventing microglial activation may be a potential therapeutic target against AD. While early recruitment of microglia is useful for clearing Aβ plaques, at later stages, it induces neuroinflammation and apoptosis.^[18]^

### 2.13. Biomarkers and their limitations (Fig. 3)

Over the years, Aβ oligomers have been recognized as promising remedial priorities for the treatment of AD. But it turns out that, in any case, many clinical examinations relying on the amyloid beta hypothesis have proved unsuccessful.^[56]^ Thus, the likelihood that many amyloid-beta proteins are no longer essential drivers of AD is enhanced. In addition, the recognition that AD is a disease caused by multiple components is gradually increasing.

**Figure 3.**
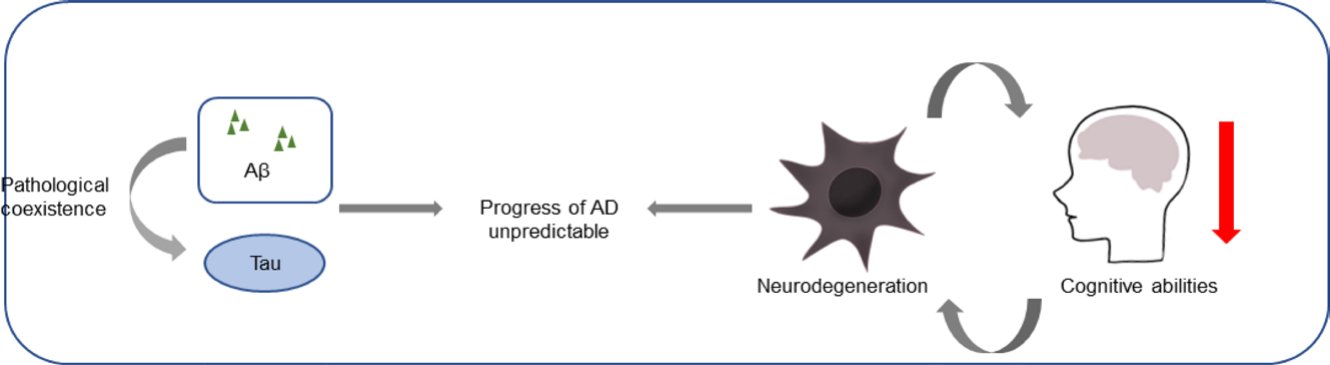
Biomarkers and their limitations.

### 2.14. The prediction accuracy of pure biological definition is low

A major limitation of the biological definition of Alzheimer’s is its low predictive accuracy. Some studies have shown^[57]^ that the presence of tau and amyloid beta positivity is insufficient to unambiguously predict the occurrence of symptoms (MCI or dementia) in individuals without clinical impairment.

### 2.15. Confusion with other diseases (Fig. 4)

Another challenge in the diagnosis of AD using pure biomarkers is that the presence of biomarkers of AD lesions in the body can be used as a primary indicator for the diagnosis of AD, as such lesions are commonly found in patients with other neurodegenerative diseases, most commonly Lewy body dementia.^[58]^ This comorbid feature of dementia with Lewy bodies in this setting confuses the acceptance of biomarker-based diagnoses. Experienced clinicians determine the pathology of Lewy body dementia based on clinical signs and symptoms (such as hallucinations and parkinsonism) or indirect biomarkers (such as DaTscan innervation) to confirm the diagnosis of Lewy body dementia.

**Figure 4.**
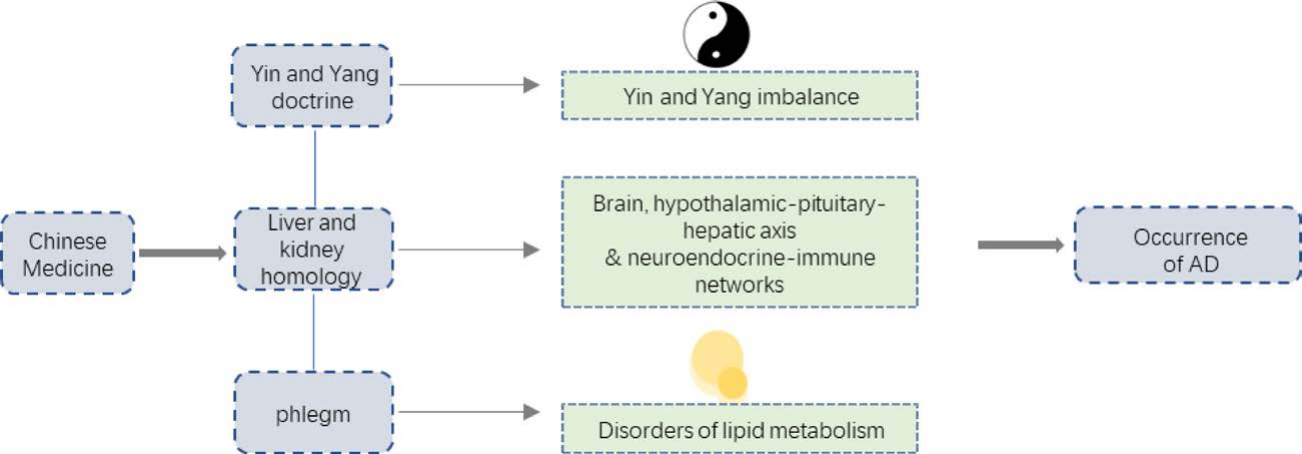
Cognition of Alzheimer’s Disease (AD) in traditional Chinese medicine (TCM).

### 2.16. Uncertainty of AD pathogenesis model

Biological models supporting the ATN classification provide the possibility of studying biological changes before the onset of symptoms, which is necessary for the development of drugs to treat the earliest stages of disease. Proponents of a biological definition of AD often refer to cancer models in which there may be a long asymptomatic phase of the disease, and all affected individuals anywhere along the disease continuum would benefit from the same treatment even in the preclinical phase.^[59]^ However, tracking positive individuals for cognitively unimpaired biomarkers claims that most of these individuals do not progress over time. At present, it is not clear whether AD is better suited to a continuum model of long-term asymptomatic prostate cancer or to a risk model in which asymptomatic amyloid-positive and tau-positive persons are at risk and persons with a clinical phenotype are in a disease state (similar to a cancer state). Therefore, defining a disease based solely on its pathological evolution rather than its clinical phenotype may lead to diagnostic bias.

In conclusion, amyloid P and Tau biomarkers are not sufficient to predict the progression of AD with confidence or to define a person’s position on the AD continuum without clinical input. The relationship between the coexistence of tau and amyloid P pathology on the one hand and the development of cognitive decline and ATN remains uncertain at the individual level.

### 2.17. Cognition of AD in TCM

This is precisely because the cognition of this disease in modern medicine is still in a relatively exploratory stage, forming a variety of hypotheses, and thus far, no research results have been widely recognized and respected by the academic community. Therefore, we should use the achievements of TCM to help us deepen the understanding and treatment of AD.

Because AD belongs to modern medical terminology, it is not recorded in classical ancient books of TCM, and later physicians classify it into different categories of TCM diseases such as “dementia,” “depression,” “mania,” “inferiority,” and even “vertigo” according to its clinical manifestations.

The etiology and pathogenesis of AD are complex, and the clinical signs and symptoms are complex and changeable; however, the basic pathogenesis is deficiency in origin and excess in superficiality. Most physicians admit that the disease is due to the patient’s old age, infirmity, deficiency of essence and blood, internal mental damage, and apraxia. It should be noted that because of the complexity of the etiology and pathogenesis of AD, TCM has formed multiple cognitions on the basis of the basic pathogenesis of this disease, and many famous experts have started from the existing theory to explore this disease.

### 2.18. The theory of yin and yang is the basis of AD

Chen Baoxin et al^[60]^ considered that the response state of microglial cells to external stimulation in modern medicine can be divided into classical activation state and selective activation state, and that the 2 states are interdependent and antagonistic. Combined with the discovery of TCM classics and the theory of yin and yang in TCM, they believed that the theory of yin and yang is the basis of AD.

Su Wen · Yin Yang Ying Xiang Da Lun^[61]^ records: “Yin and yang mutually interact.” The discipline of all things Treatment must be based on the root. Everything is divided into yin and yang, which are mutually opposed, mutually restricted, mutually rooted, and mutually used. The so-called “Balance between yin and yang.”^[61]^ If yin and yang are unbalanced, all diseases will occur. The imbalance between yin and yang is also essential for the occurrence and development of this disease. AD patients will experience memory loss, cognitive behavior disorders, depression, and other symptoms, which are due to the patient’s yang deficiency, yang damage to yin. There is a saying in Lei Zheng Zhi Cai^[62]^: “Some patients with this disease are lethargic during the day and hyperactive at night, which is caused by the deficiency of yang qi, the hyperactivity of yin qi, the failure of yang to enter yin and the failure of yang to control yin.” Therefore, in order to treat this disease in TCM clinics, we must take yin and yang as the guiding principles, observe yin and yang carefully, and keep the pathogenesis.

### 2.19. The theory of “liver and kidney share the same source” and AD

The theory of the liver and kidney is an important part of the theory of visceral manifestation, which was also derived from Neijing and carried forward by later generations. Su Wen · Wu Yun Da Lun^[63]^: “Salty generates the kidney, the kidney generates the bone marrow, and the marrow generates the liver.” The liver and kidney are homologous; that is, the liver stores blood, the kidney stores essence, and the liver and kidney are homologous; that is, the essence and blood are homologous.

Yufang et al^[64]^ treated AD based on the theory of liver and kidney homology. According to Wang Qingren,^[65]^ “Inspiration and memory are in the brain, not in the heart.” In Compendium of Materia Medica,^[66]^ it is said that “the brain is the house of the primordial spirit..” Miraculous Pivot: Meridians^[61]^ states, “The kidney stores the essence and produces the marrow, which passes through the brain..” If the kidney is damaged, the kidney does not store essence, and the kidney does not produce marrow, the brain and marrow will be deficient, the mind will be out of use, memory will be reduced, and the spirit will be exhausted. The liver and the kidney are of the same origin, the mother and the child are mutually generated, and the kidney qi is full, so the liver blood is full, and the liver qi is smooth; If the liver fails to regulate the flow of qi, the disease of the child will affect the mother, causing the kidney to fail to store essence, and the marrow will strengthen the brain and disappear, resulting in dementia, depression, vertigo, and other syndromes.

Speaking of the impact of the theory of liver and kidney homology on the treatment of AD, we have to mention that Li Hanmin^[67]^ and others found that bone marrow mesenchymal cells can be induced to differentiate into stem cells through modern experiments, which confirmed the hypothesis that “kidney dominates bone and marrow, marrow and liver,” and on this basis. The theory that “liver and kidney are homologous to essence and blood” was further developed into the theory that “liver and kidney are homologous to brain, hypothalamus-pituitary-liver axis, and neuro-endocrine-immune network”,^[67]^ which provided modern medical proof for the theory of “liver-kidney homology.” Since then, many animal experiments have been conducted to study the possibility of treating AD based on the theory of “liver-kidney homology.”

### 2.20. Treatment of AD from phlegm

Min Dongyu et al,^[68]^ based on the fact that modern medical research found that abnormal lipid metabolism was closely related to the occurrence and development of AD, combined with the theory of TCM, believed that “phlegm evil” was the key factor in the onset of AD and advocated for treating AD from phlegm.”

TCM believes that lipids belong to body fluids, and lipid transfer is not conducive to the development of hyperlipidemia. Dyslipidemia is closely related to atherosclerosis, a high-risk factor for AD. Duan Li^[69]^ and other studies have proved that lipid metabolism disorder is an important material basis for the formation of “phlegm evil,” and will affect the changes of phospholipid composition in AD brain tissue, thus affecting nerve function.

Since Synopsis of Prescriptions of the Golden Chamber, there is a saying in TCM that “all diseases are caused by phlegm” and “strange diseases are caused by phlegm.” There is a saying in Danxi Xinfa^[70]^: “There are many people who are forgetful and short of spirit, and there are also people with phlegm.” In Shishi Mi Lu,^[71]^ “Phlegm is the most abundant, and dull is the deepest.” These findings suggest a relationship between phlegm and dementia.

TCM believes that phlegm causes AD because it covers the heart and causes apraxia, which leads to dementia. On one hand, phlegm evil and blood stasis block the brain orifices, and the primordial spirit loses nourishment, such as infatuation. In addition, all diseases are caused by phlegm, and phlegm evil is the pathological product of the joint action of multiple viscera dysfunction, which is called by the ancients that “the spleen is the source of phlegm, the lung is the storage vessel of phlegms, the liver is the vessel of wind phlegms, and the kidney is the root of phlegms. Therefore, phlegm-evil disease is complex and multi-terminal, and phlegm-evil stasis obstruction, phlegm- caused stasis, can form the pathological state of.” Phlegm and blood stasis in the same disease, phlegm clouding the mind, and blood stasis blocking the brain orifices cause the onset of lingering and repeated AD, forming the syndrome of deficiency in origin and excess in superficiality.

In addition, there are also the treatment of AD from the perspective of “brain spirit”^[72]^ and the treatment of AD from the perspective of “microorganism-intestine-brain axis”,^[73]^ all of which have made noble and gratifying research on the cognitive treatment of AD in TCM.

### 2.21. Treatment of AD by acupuncture and medicine

On October 11, 2019, the World Federation of Chinese Medicine Societies and the Chinese Society of TCM jointly issued the International Guidelines for Clinical Practice of TCM for Alzheimer’s Disease (2019-10-11).^[74]^ Based on the Guidelines for Clinical Diagnosis and Treatment of Alzheimer’s Disease in Internal Medicine of TCM (Standard No.: T/CACM1315-2019) issued by the Chinese Society of TCM in 2019, and in combination with clinical research in recent years, the guidelines were formed through expert discussion, with a view to standardizing academic research on AD in TCM. To guide clinical practice.

The guideline standardizes the diagnosis of AD in TCM, that is, a mental disorder caused by marrow reduction, brain elimination and mental apraxia, with the main clinical manifestations of stupidity, mental retardation and forgetfulness, which belongs to “dementia” in TCM. The disease is divided into early, middle, and late stages: marrow sea empty, spleen and kidney yang deficiency, liver and kidney yin deficiency, yin deficiency, and fire hyperactivity.

In this study, according to the guidelines of TCM syndrome differentiation, combined with acupuncture theory, we explored the combination of acupuncture and medicine in the treatment of AD.

## 3. Treatment based on pattern identification in TCM (Fig. 5)

### 3.1. The sea of marrow is empty

Patients of this type experience memory loss, listlessness, dizziness and tinnitus, slow action, lassitude and lethargy, hair loss and teeth loss, pale red or pale tongue, and thready or deep and thready pulse. It should be used to nourish the liver and kidneys, replenish the essence, and produce marrow. The basic prescription was Qifu Yin or Guilu Erxian Jiao.

**Figure 5.**
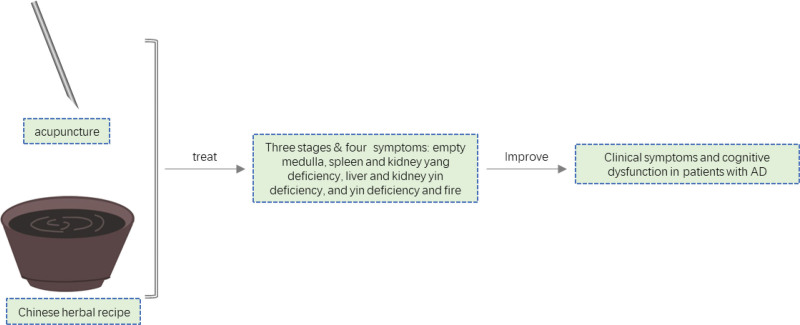
Traditional Chinese medicine (TCM) treatment based on syndrome differentiation.

According to relevant animal experiments, the Tibetan medicine Ershiwuwei Shanhu Pill has a significant effect on the treatment of AD.^[75]^ Xue Jing et al^[72]^ believed that this syndrome should be treated with modified Dihuang Yinzi to generate the essence and marrow, and enrich the brain. Lin Yudong et al^[76]^ studied the treatment of AD patients with kidney deficiency and marrow deficiency by Bushen Yisui Fang (Epimedium, Psoralea corylifolia, Polygonum multiflorum, Ligustrum lucidum, Ligusticum chuanxiong and Acorus tatarinowii), and made gratifying achievements. Yucheng et al^[77]^ used a prescription for tonifying the kidney and replenishing the marrow, which was composed of Rhizoma Acori Tatarinowii, Radix Polygalae, Radix Polygoni Multiflori, Herba Epimedii, Carapax et Plastrum Testudinis, and Os Draconis, to conduct a randomized controlled trial and found that this prescription could improve the learning and memory ability of AD rats, and its mechanism may be related to the improvement of mitochondrial oxidative stress. Shenggeli et al^[78]^ found that Yizhi Zhidai Prescription (Radix Rehmanniae Preparata, Rhizoma Chuanxiong, Rhizoma Acori Tatarinowii, Fructus Corni, Fructus Alpiniae Oxyphyllae, Radix Polygalae, Radix Astragali, Pheretima, Colla Cornus Cervi, Radix Angelicae Sinensis, Radix Curcumae, Hirudo, and Radix et Rhizoma Rhei) combined with donepezil can significantly improve the memory function and daily living ability of AD patients to improve the levels of serum acetylcholin esterase and CAT, and reduce the incidence of adverse reactions. Based on evidence-based medicine, Shi Jing et al^[79]^ systematically evaluated the treatment of AD with the method of tonifying the kidney, and concluded that the method of tonifying the kidney and invigorating the spleen was beneficial to the cognition of AD patients, the method of reinforcing the kidney and resolving phlegm, and the method for tonifying the kidney and activating blood circulation combined with western medicine had a synergistic effect on the cognitive function and daily living ability of AD, and the method for tonifying the kidney and activating blood circulation was beneficial to the mental and behavioral symptoms of AD.

### 3.2. Yang deficiency of spleen and kidney

Patients with this type have reduced diet, anorexia, abdominal distension, soreness and weakness of the waist and knees, clear and long urine, loose stool, aversion to cold and weakness, pale, fat, and tender tongue with teeth marks on the edge, and deep, slow, and weak pulse. It should be treated by tonifying the spleen and kidneys, nourishing the body, and tranquilizing the mind. The foundation was Shaodan.

As a traditional Tibetan medicine, Sanwei Cardamom Decoction has the effect of tonifying the spleen and kidney, calming the heart, and tranquilizing the mind, and is widely used in the treatment of primary insomnia, AD, and other mental diseases. Animal experiments have shown that the Tibetan medicine Sanwei Doukou Decoction^[80]^ can significantly improve the memory ability of AD model mice, promote the survival of neurons, and reduce the loss of functional neurons.

Huang Binsen et al^[81]^ conducted a randomized controlled trial using the Yijing Jiannao Prescription. Rhizoma Polygonati, Radix Astragali, Radix Rehmanniae Preparata, and Cortex Eucommiae can tonify the kidney and spleen, Fructus Amomi can invigorate the spleen and eliminate dampness, Fructus Alpiniae Oxyphyllae can warm the kidney and arrest nocturnal emission, Radix Polygoni Multiflori can tonify essence and blood, and Fructus Schisandrae enters the liver and tonifies the clinical curative effect. Wang Yanxin et al^[82]^ used Wuzi Yanzong Pill and Xixin Decoction to treat AD in the spleen and kidney, and achieved good results in clinical practice.

### 3.3. Yin deficiency of liver and kidney

Patients with this type have hypomnesis, dull expression, depression, quiet and solitary, vertigo and tinnitus, numbness of limbs, soreness and weakness of the waist and knees, dark red tongue, thin and small tongue body, thin and white or little tongue coating, deep and thready pulse, or deep and thready pulse. It should be treated by tonifying the liver and kidney, nourishing yin, and suppressing yang. Zuoguycline should be used as a basic prescription.

Wu Chao et al^[83]^ a randomized controlled trial was conducted to treat AD of liver and kidney deficiency with Bugan Zhuangshen Fang (basic prescription: Radix Polygoni Multiflori Preparata, Fructus Corni, Rhizoma Dioscoreae, Radix Rehmanniae Preparata, Semen Cuscutae, Fructus Mori, and Carapax et Plastrum Testudinis) combined with Western medicine, compared to donepezil hydrochloride alone in the control group. The MMSE score and C-reactive protein and homocysteine levels in the study group were significantly improved (all *P* < 1.05), whereas there was no significant change in the control group (all *P* > .05), and the study group was significantly better than the control group (all *P* < .05).

Based on the theory of “correlation between liver and kidney,” Chunlin et al^[84]^ discussed the treatment of AD in liver and kidney deficiency with Heixiaoyao San (Radix Rehmanniae Preparata, Radix Bupleuri, Radix Angelicae Sinensis, Radix Paeoniae Alba, Poria, Rhizoma Atractylodis Macrocephalae, Herba Menthae, Rhizoma Zingiberis Recens, and Radix Glycyrrhizae Preparata) from the Ca2+ -CaM/CaMKⅡ-CREB signal transduction pathway. Wang Ruqian believes that this prescription is most suitable for the elderly and infirm with a deficiency in both the liver and kidney. All medicines in the prescription are used together; the liver, kidney, and spleen are regulated together, and qi, blood, and essence are considered together, so that the effects of soothing the liver, tonifying the kidney, dredging collaterals, and benefiting orifices are achieved. Heixiaoyao Powder is in line with the research idea of treating AD in the liver and kidney. Simultaneously, the Ca2+ -CaM/CaMKII-CREB signaling pathway is closely related to the pathogenesis of AD. Based on this, Ma Chunlin et al^[85]^ carried out an animal intervention experiment with Heixiaoyao Powder and achieved the expected verification.

Yuan Xiuli^[86]^ is good at treating AD by combining acupuncture and medicine with the method of “soothing the liver, tonifying the kidney and regulating the spirit,” and believes that each of the 5 internal organs has its own will, and the disorder of the 7 emotions and 5 emotions hurts the internal organs, so besides acupuncture, it is also necessary to cooperate with emotional therapy to enlighten patients, carry out rehabilitation training, eat reasonably, ensure adequate sleep, and cultivate interest, so as to help treat diseases. Acupuncture prescription (tonifying kidney and regulating mind): Dazhong, Sishencong, Shuigou, Shenmen, Sanyinjiao, Taichong, and Hegu. TCM prescriptions (soothing the liver and tonifying the kidney): Radix Astragali, Rhizoma Chuanxiong, Flos Carthami, Radix Paeoniae Rubra, Radix Bupleuri, Fructus Aurantii, Pericarpium Citri Reticulatae, Succinum, Semen Ziziphi Spinosae, Poria, Radix Polygalae, Rhizoma Acori Tatarinowii, and Scorpio. TCM is combined with acupuncture manipulation to play a remarkable role.

### 3.4. Hyperactivity of fire due to yin deficiency

Hypomnesia, palpitations, insomnia, hectic fever, night sweats, feverish sensation in the chest, palms, and soles, red eyes, red cheeks, nocturnal emission, tinnitus, soreness and pain of the waist and knees, scanty urination, constipation, red tongue, lack of body fluid, and thready and rapid pulse. It should be treated by nourishing the yin and blood, clearing the liver, and purging the fire. The basic prescription should be Huanglian Jiedu Decoction combined with Tianwang Buxin pills.

Li et al^[87]^ conducted a randomized controlled trial to test the efficacy of blood-nourishing brain-clearing granules in the treatment of AD and its effect on vascular endothelial growth factor. The prescription of blood-nourishing brain-clearing granules comprises prepared rehmannia root, Chinese angelica, Szechuan lovage rhizome, white paeony root, gambir plant, suberect Spatholobus stem, common self-healing fruit-spike, cassia seed, nacre, yanhusuo, and Manchurian wildginger. It has the efficacy of nourishing blood, clearing away heat, promoting blood circulation, dredging collaterals, and tonifying the liver and kidney, and can improve intracerebral microcirculation disturbance. The results show that Yangxueqingnao granules have a significant effect on AD patients, can effectively improve cognitive function, and are safe.

Li et al^[88]^ Liuwei Dihuang Pill was combined with conventional Western medicine to carry out clinical observations on the treatment of mild and moderate AD patients with hyperactivity due to yin deficiency. Liuwei Dihuang Pill is a famous prescription for nourishing yin and purging fire, tonifying the kidney, and replenishing essence, which plays an increasingly important role in the treatment of AD. The results showed that the total effective rate of the combination group was significantly higher than that of the control group (*P* < .05). Conclusion: Liuwei Dihuang Pill combined with conventional Western medicine for the treatment of mild-to-moderate AD can significantly improve the intelligence and living ability of patients, which is worthy of clinical application.

### 3.5. Acupuncture treatment

Acupuncture plays a vital role in the treatment of AD, and its importance is even greater than that of TCM. Therefore, in the process of TCM diagnosis and treatment of AD, acupuncture should not be placed at the subsidiary level of syndrome differentiation and treatment of TCM, and the combination of acupuncture and medicine should not be acupuncture combined with TCM; the two should be in the same position, pay equal attention to acupuncture and medicine, and complement each other.

As an important part of TCM, acupuncture and moxibustion play an important role in the treatment of AD. According to basic medical research, the mechanism of action of acupuncture and moxibustion on AD^[89]^ includes the following aspects: regulation of the expression of related proteins, inhibition of central inflammatory response, resistance to oxidative stress injury, regulation of energy metabolism in brain regions, improvement of synaptic plasticity of neurons, regulation of the activity level of autophagy, and inhibition of nerve cell apoptosis. This article discusses the treatment of AD using acupuncture.

According to the latest clinical guidelines, AD can be divided into 4 syndromes: emptiness of the sea of marrow, yang deficiency of the spleen and kidney, yin deficiency of the liver and kidney, and fire hyperactivity due to yin deficiency. On this basis, according to the relevant complex network analysis^[90]^ and guidelines recommendation,^[74]^ the main points of acupuncture prescription are Baihui, Sishencong, Zusanli, Shenmen, Neiguan, Taixi, Sanyinjiao, Shenting, etc. Acupoints can be used for treatment based on syndrome differentiation and can be added or subtracted according to symptoms. Shenshu and Xuanzhong can be used to treat the deficiency of Suihai; Spleen and kidney yang deficiency can be combined with Pishu, Xuehai, and Ganshu. Yin Ling Quan, Ganshu, and Sanyinjiao can be added to patients with liver-kidney yin deficiency; Sanyinjiao, Zusanli, Yinlingquan, Taixi, and Taichong can be added to patients with hyperactivity due to yin deficiency.

Wang Zidong et al^[91]^ conducted animal experiments on mice using the electroacupuncture method of “Tongdu Qishen.” In the electroacupuncture group, the Renzhong acupoint was punctured, the Baihui and Yintang acupoints were punctured horizontally according to Experimental Acupuncture and Moxibustion, and the needle handle was connected to an electroacupuncture instrument for 15 min. In the control group, donepezil was administered via gavage. The results showed that “Tongdu Qishen” electroacupuncture could improve the learning and memory function of SAMP8 mice and improve the cognitive ability of mice.

Li Ran et al^[92]^ conducted research on the intervention of acupuncture in SAMP8 mice with AD, and the research showed that acupuncture could intervene in the generation and clearance of beta-amyloid protein and the phosphorylation of Tau protein, improve mitochondrial and synaptic structure, enhance autophagy activity, intervene in the pathological process of AD, and improve memory and cognitive function in mice.

Songjiang et al^[93]^ designed animal experiments and randomly divided AD model mice into electroacupuncture group and model groups. According to Experimental Acupuncture and Moxibustion, the mice were given electroacupuncture stimulation, and the acupoints were “Baihui,” “Fengfu, and “Shenshu.” The experimental results concluded that electroacupuncture intervention could effectively prevent memory decline in AD model mice and regulate the differential protein expression caused by AD.

According to the literature on animal experiments in the past 5 years, acupuncture and moxibustion can improve the behavioral and biochemical indices of AD model animals, which is effective and safe.

### 3.6. Combination of acupuncture and medicine

According to the findings of relevant studies,^[93]^ the combination of acupuncture and medicine can achieve more significant effects than simple acupuncture or simple TCM and Western medicine and has fewer side effects. Therefore, to a certain extent, the combination of acupuncture and medicine represents the future direction of TCM in the treatment of AD.

Song Guangjie et al^[94]^ used Tongqiao Quyu Yin (Radix Astragali, Rhizoma Chuanxiong, Radix Paeoniae Rubra, Radix Angelicae Sinensis, Rhizoma Acori Tatarinowii, Semen Persicae, Radix Puerariae, Radix Curcumae, Radix Achyranthis Bidentatae, Radix Platycodonis, and Radix Glycyrrhizae Preparata) combined with scalp acupuncture (Baihui, Benshen, Shenting, Taixi, Fenglong, Sanyinjiao, and Zusanli To observe the therapeutic effect on AD patients and the influence on the indexes of inflammatory factors and peroxidation damage. The results showed that this treatment could significantly improve the level of intelligence and memory of patients, smooth their emotions, improve their quality of life, and significantly reduce inflammatory and peroxidative damage.

Li Jiamei et al^[95]^ conducted animal experiments on AD mouse models using acupuncture and medicine based on the theory of “mutual assistance” of the kidney and brain. According to Experimental Acupuncture and Moxibustion, Baihui, Shenshu and Sanyinjiao were selected for acupuncture, and Bushen Huoxue Decoction (Salvia miltiorrhiza, Ligusticum wallichii, Dioscorea opposita, Rehmannia glutinosa, Antler Glue, Lycium barbarum, Achyranthes bidentata, Cuscuta chinensis). The results showed that acupuncture combined with medicine had a positive effect on the regulation of Aβ and Tau protein in SAMP8 mice and significantly reduced the content of Aβ and P-tau protein in the cerebral cortex, so as to achieve the purpose of treating AD.

Wang Yuehua et al^[96]^ carried out clinical observations of AD based on Qin’s “Touba Needle” and the combination of acupuncture and medicine. The main points of the experiment were Baihui, Yintang, bilateral Toulinqi, bilateral Shuigu, and bilateral Feng Chi., bilateral Hegu (LI 4), bilateral Quchi (LI 11), bilateral Zusanli (ST 36), and bilateral Taichong (LR 3) points, combined with Donepezil Hydrochloride Tablets, can effectively improve cognitive impairment in AD patients and enhance their self-care ability and social adaptability.

Wang et al^[97]^ observed the clinical effects of Dihuang Yizhi Fang, electroacupuncture, and Donepezil Hydrochloride Tablets in the treatment of AD. The observation group was treated with Dihuang Yizhi Fang (Radix Rehmanniae Preparata, Fructus Alpiniae Oxyphyllae, Radix Salviae Miltiorrhizae, Rhizoma Acori Tatarinowii, Colla Plastri Testudinis, Poria, Placenta Hominis), electroacupuncture (Baihui, Shenting, Fengfu, and Mingmen), and donepezil hydrochloride, which could effectively improve the short-term therapeutic effects of AD patients with AD.

Li Haijun et al^[98]^ carried out clinical observations to discuss the treatment of AD using Tongqiao Huoxue Decoction combined with Huiyang Nine Needles. In the experimental group, Tongqiao Huoxue Decoction was a famous prescription created by Wang Qingren through the ages, and the main points of the Huiyang Nine Needles were Taixi, Sanyinjiao, Laogong, Yongquan, Hegu, Sanli, Yamen, Huantiao, and Zhongwan. Olanzapine was administered orally to the control group. The results showed that the effect of Tongqiao Huoxue Decoction combined with Huiyang Jiuzhen was similar to that of olanzapine, but the former had higher safety and lower incidence of adverse reactions.

Wang Xinyu et al^[99]^ observed the clinical effect of Xingshen Kaiqiao acupuncture (the main points are Temporal Three Needles, Brain Three Needles, Sishen Needles, Zhisan Needles, and Shenmen) combined with Maixuekang Capsules, and found that it could effectively improve the clinical signs, symptoms, and cognitive dysfunction of AD patients and had good anti-dementia effects.

## 4. Conclusion

Although a purely biological definition of AD has been proposed in the past, with the progress of research, an increasing number of studies have pointed out that amyloid P and Tau biomarkers are not enough to predict the progress of AD with confidence, nor are they enough to be applied to the clinical environment and diagnosis of people without cognitive impairment. The relationship between the coexistence of tau and amyloid P pathology on the one hand and the development of cognitive decline and ATN remains uncertain at the individual level.

In the past 20 years, the number of deaths related to AD has increased significantly, but the treatment of Western medicine remains vague. FDA-allowed treatments for AD have not shown any remedial effects and have not been able to reduce the rate of disease progression. Most of the drugs recommended for use are acetylcholinesterase inhibitors and NMDA receptor antagonists. However, none of the potential drugs developed have been successful beyond phase II/III trials, and the challenges remain low bioavailability, prolonged serum half-life, and inability to cross the blood-brain barrier.

TCM therapy AD has unique advantages and is increasingly being widely recognized by the international community, and related studies have emerged endlessly at home and abroad.^[100]^ TCM, acupuncture, and moxibustion, especially the combination of acupuncture and medicine, play vital roles in the treatment of AD. Acupuncture and moxibustion, as ancient treatment methods, can dredge meridians, regulate qi and blood, replenish essence and marrow, dredge the governor vessel, regulate the spirit, and tonify the kidney by needling relevant acupoints. TCM treatment of AD has few side effects and can play a lasting advantage. Based on the theory of visceral manifestation of TCM, syndrome differentiation, and treatment, its mechanism of action is to stimulate the cerebral cortex to synthesize nicotinic ACH, reduce blood lipids, dilate cerebral vessels, increase cerebral blood flow, and improve cerebral inflammatory injury.

The combination of acupuncture and medicine combines the advantages of TCM and acupuncture, exerts a combined therapeutic effect of drugs and acupoint stimulation, and treats both symptoms and root causes. This has been explained in detail before the article and is not repeated here.

TCM therapy for AD mostly involves compound treatment and multi-point acupuncture treatment, and its mechanism of action is not yet clear. Although in recent years, the Chinese medicine community has made more and more gratifying research, it still needs more relevant basic research, animal experiments, and clinical observations to explain and clarify the strategy of TCM therapy for AD.

In addition, the study of AD in TCM is closely followed by modern medicine. After absorbing the frontier achievements of modern medicine, it has been applied to the study of TCM. We really lack research based on the tradition of TCM, based on classical ancient books, mainly on TCM, absorbing the advanced achievements of modern medicine for our use, and the times call for us to regain the characteristics of TCM.

## Author contributions

**Conceptualization:** Zhao Liu.

**Data curation:** Zhao Liu, Ruiqian Guan, Fan Bu, Limin Pan.

**Formal analysis:** Zhao Liu.

**Funding acquisition:** Zhao Liu.

**Investigation:** Zhao Liu.

**Methodology:** Zhao Liu.

**Project administration:** Zhao Liu.

**Resources:** Zhao Liu.

**Software:** Zhao Liu.

**Supervision:** Zhao Liu.

**Validation:** Zhao Liu.

**Visualization:** Zhao Liu.

**Writing – original draft:** Zhao Liu.

**Writing – review & editing:** Zhao Liu.
